# Understanding the Morphological Evolution of InSb Nanoflags Synthesized in Regular Arrays by Chemical Beam Epitaxy

**DOI:** 10.3390/nano12071090

**Published:** 2022-03-26

**Authors:** Isha Verma, Valentina Zannier, Vladimir G. Dubrovskii, Fabio Beltram, Lucia Sorba

**Affiliations:** 1 NEST, Scuola Normale Superiore and Nanoscienze-CNR, Piazza San Silvestro 12, I-56127 Pisa, Italy; isha.verma@sns.it (I.V.); fabio.beltram@sns.it (F.B.); lucia.sorba@nano.cnr.it (L.S.); 2 Faculty of Physics, St. Petersburg State University, Universitetskaya Emb. 13B, 199034 St. Petersburg, Russia; dubrovskii@mail.ioffe.ru

**Keywords:** InSb nanoflags, InP nanowires, chemical beam epitaxy, regular array, growth modeling

## Abstract

InSb nanoflags are grown by chemical beam epitaxy in regular arrays on top of Au-catalyzed InP nanowires synthesized on patterned SiO_2_/InP(111)B substrates. Two-dimensional geometry of the nanoflags is achieved by stopping the substrate rotation in the step of the InSb growth. Evolution of the nanoflag length, thickness and width with the growth time is studied for different pitches (distances in one of the two directions of the substrate plane). A model is presented which explains the observed non-linear time dependence of the nanoflag length, saturation of their thickness and gradual increase in the width by the shadowing effect for re-emitted Sb flux. These results might be useful for morphological control of InSb and other III-V nanoflags grown in regular arrays.

## 1. Introduction

Low-dimensional InSb nanostructures have sparked interest in the past few years due to their potential applications in high-speed and low-power electronics [1,2], infrared optoelectronics [3], spintronics [2,4,5], quantum electronics [6,7], and topological quantum computation [8]. These applications stem from the outstanding intrinsic properties of InSb such as a narrow band gap (≅0.23 eV) [4,9,10], high bulk electron mobility (7.7 × 10^4^ cm^2^/(V s)) [1,11], small effective mass (*m_∗_* = 0.018 m_e_) [4,11,12,13,14,15], and a large Landé g-factor (*|g_∗_|*∼50, [11,15]). Among the most influential developments are the topological superconducting quantum devices based on InSb nanowires (NWs) [16,17].

Besides one-dimensional NWs, quasi two-dimensional (quasi-2D) InSb nanostructures, often called nanoflags (NFs), also attract great attention owing to their inherent design flexibility [4,9,15,18]. InSb NF-based devices have been proven appropriate for studies of novel quantum phenomena, development of scalable topological superconducting devices based on strong spin−orbit coupling [19,20,21], and infrared (IR) photodetectors exhibiting a broad spectral detection range [22]. These devices require high crystal quality and dimensional precision. Therefore, a deep understanding of the growth mechanisms and the morphology evolution of such NFs is really crucial. Growth modeling is also fundamental for the controlled growth of NFs with the desired morphological properties and crystal structure, similarly to the growth theory of III-V NWs [23,24,25,26,27,28,29,30,31,32].

Recently, De La Mata et al. [11] and Gazibegovic et al. [33] reported that a single twin plane drives the crystal to change its geometry and expand, leading to asymmetric NF morphology, while Pan et al. [34] attributed the NF formation mechanism to a combination of the vapor−liquid−solid (VLS) axial growth and the vapor–solid (VS) lateral growth. However, the main focus of these works was more on tailoring the growth parameters to obtain the maximized lateral dimension, rather than on understanding the morphology evolution of the NFs. In our previous studies [18,35], we were focused on the development of a growth approach that allowed us to obtain InSb NFs with the maximized elongation keeping the minimum thickness. These NFs exhibited high crystal quality and high electron mobility. A detailed study of Gazibegovic et al. [33] provided an explanation of the formation mechanism and morphology of a single InSb NF related to the substrate design and growth condition. However, the growth modeling of regular arrays of NFs influenced by the shadowing effect in the directional deposition techniques such as chemical beam epitaxy (CBE) has not been presented so far.

In this work, we investigate the growth mechanisms of InSb NFs in more detail. We analyze the shape evolution of Au-catalyzed InSb NFs on InP NW stems grown in regular arrays on lithographically patterned InP(111)B substrates using a combined selective area (SA) and vapor–liquid–solid (VLS) growth. We propose a model describing the InSb NF growth and morphology as a function of time and pitch of the NW/NF array. By fitting the experimental data, we are able to deduce the most important parameters influencing the width and thickness of InSb NFs.

## 2. Materials and Methods

InP-InSb NFs were synthesized by CBE in a Riber Compact-21 system on InP(111)B substrates via Au-assisted SA growth. A 20 nm-thick sputtered SiO_2_ was used as a mask on InP(111)B to suppress the parasitic growth on the substrate surface. The openings in the SiO_2_ mask were made by wet etching (HF) on hexagonal arrays of lithographically patterned substrate with different spacing in <112> direction, which we refer to as the pitch a, followed by 6 nm-thick Au evaporation and lift-off. This resulted in a hexagonal array of Au discs of 30 ± 3 nm in diameter, inside SiO_2_ mask openings of 153 ± 7 nm in diameter, positioned at a fixed distance (200 nm) in <110> direction, and different pitches from 500 nm to 1500 nm in the <112> (perpendicular) direction. The corresponding range of Au disc density varied from 3.6 to 12.3 μm^−2^. The hexagonal geometry of the arrays is determined by the processing technique, and no effect of the fixed spacing in the <110> direction is addressed in what follows. These Au discs catalyzed the CBE growth of InP NWs and InSb NFs. Trimethylindium (TMIn), tert-butylphosphine (TBP), trimethylantimony (TMSb) were used as metal-organic precursors.

InP NW stems were grown for 60 min under TMIn and TBP line pressures of 0.6 and 1.2 Torr respectively, at a growth temperature T_InP_ of 405 °C ± 5 °C, as measured by a pyrometer. For InP NW growth, the sample was rotated at 5 rotations per minute. The InSb segments were grown on top of these InP stems without rotation of the substrate. The alignment of InSb NFs was achieved using reflection high-energy electron diffraction (RHEED) pattern, following the same procedure as reported in Ref. [18]. The substrate was aligned in such a way that the pitch direction was parallel to the projection of Sb beam. Afterwards, the substrate temperature was ramped down under TBP flux, to the optimized InSb growth temperature (ΔT = −40 °C with respect to T_InP_).

For initiation of InSb growth, group V flux was abruptly switched from TBP to TMSb. InSb NFs were grown at a constant temperature, with TMIn line pressure of 0.6 Torr and TMSb line pressure of 1.2 Torr, for different times. At the end of growth, the samples were fast cooled down to room temperature in ultra-high vacuum (UHV) environment in the absence of group V flux in order to prevent the accumulation of Sb on the sidewalls of InP-InSb heterostructures.

Morphological characterization of the samples was performed using field emission scanning electron microscopy (SEM) with a Zeiss Merlin SEM operating at an accelerating voltage of 5 keV.

## 3. Results and Discussion

In order to study the effect of the pitch on the morphology of InP-InSb heterostructured NFs, we fabricated many patterns with different pitches on the same substrate. Hence, the same growth occurred simultaneously on the patterns with different densities of Au discs corresponding to the pitches of 500, 700, 900, 1100, and 1500 nm. Using SEM images, we measured the lengths L, widths W, and thicknesses T of InSb NFs obtained on the patterns with different pitches a and after different InSb deposition times t.

**Figure 1:**
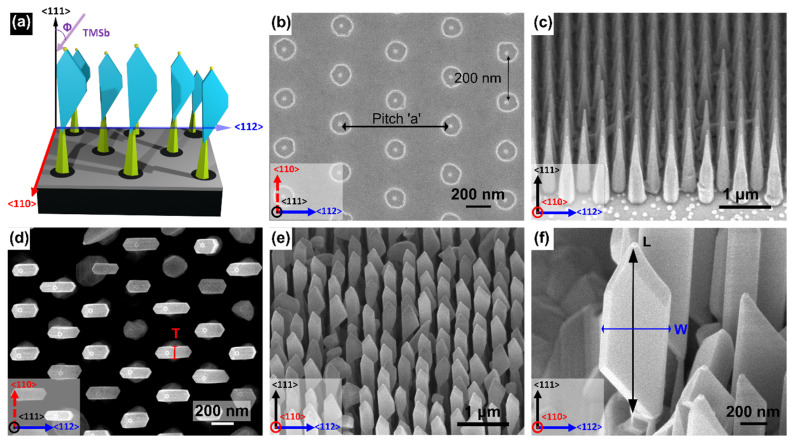
(**a**) Three-dimensional representation of the directional growth configuration with TMSb injector forming an angle Φ of 38° with the normal substrate direction <111>. The in-plane Sb beam projection is aligned with the pitch direction <112>. (**b**) Top-view SEM image of a lithographically patterned InP(111)B substrate with 20 nm-thick SiO_2_ mask and Au discs having a pitch a of 700 nm. (**c**) 45° tilted SEM image of InP NW stems grown for 60 min on the substrate shown in panel (**b**). (**d**) Top-view and (**e**) 45° tilted SEM image of InP-InSb NFs obtained after 60 min growth of InSb. (**f**) Magnified 45° tilted SEM image of an individual InSb NF. Panels (**d**,**f**) show the measured geometrical parameters of InSb NFs: thickness T was measured using top-view images, width W and length L were measured using tilted images and corrected by the geometrical factor due to the tilt angle. The crystallographic directions are given at the bottom left corner of the panels.

Figure 1a shows 3D representation of the growth configuration with TMSb injector at an angle Φ of 38° with the normal substrate direction <111>. The in-plane TMSb beam projection is aligned with the pitch direction <112>. Figure 1b shows top-view SEM image of a patterned substrate with 700 nm pitch. InP NW stems were grown in regular array on the lithographically defined substrate as shown in Figure 1c. These NWs have a length of 1.2 ± 0.1 μm, a tip diameter of 46 ± 5 nm, a base diameter of 238 ± 35 nm, and an Au nanoparticle diameter of 38 ± 2 nm. The dimensions mentioned are the average values and the standard deviations. InSb NFs were grown on top of the InP NW stems. The top view and 45° tilted SEM images of InSb NFs are shown in Figure 1d,e, respectively. NFs with different number of sidewalls and aperture angles (i.e., the angle at the base between the InP NW stem and the NF) were obtained, as was already discussed in [35]. Figure 1f shows the magnified image of a representative NF.

It is worth mentioning that even with the use of SiO_2_ mask for SA CBE growth, nucleation and growth of parasitic islands is observed on the mask surface for all growth times. We assume this to be due to a non-perfectly homogeneous SiO_2_ layer. Hovewer, the yield of NWs or NFs is not compromised, being always higher than 85%. A low magnification top-view SEM image of InP-InSb heterostructure NFs grown for 60 min in the pattern with a pitch a of 700 nm is shown in Figure S1 of Supplementary Materials.

**Figure 2:**
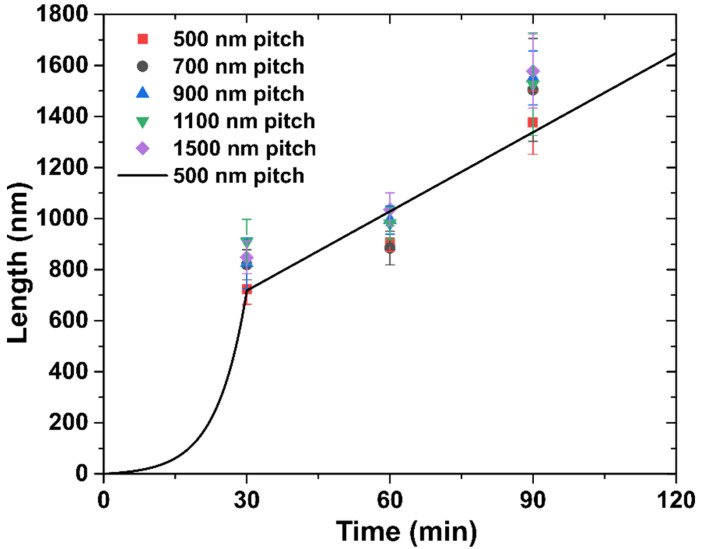
Dependence of the length of InSb NFs on the growth time for different pitches shown in the legend. Line shows the fit for 500 nm pitch.

**Figure 3:**
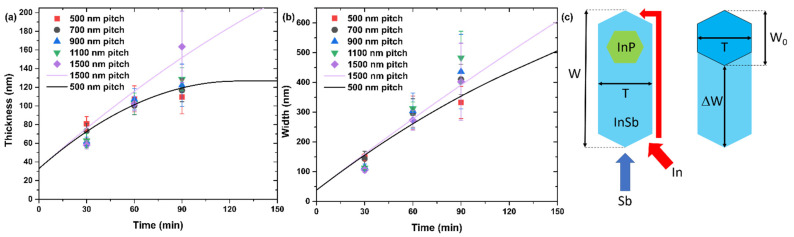
(**a**) Width W and (**b**) thickness T of InSb NFs versus growth time for different pitches a shown in the legend. (**c**) Illustration of the NF geometrical parameters from top view used in the model. Lines in (**a**,**b**) show the fits for pitches a = 1500 and 500 nm obtained within the model.

The measured dependences of the length L, width W, and thickness T of InSb NFs on the growth time are shown in Figure 2 and Figure 3a,b, respectively, for different pitches from 500 to 1500 nm. Non-linear length evolution shown in Figure 2 can be qualitatively explained by considering the In-limited VLS axial growth rate containing two contributions: (1) the direct impingement and (2) In adatom diffusion on the NF sidewalls. Surface diffusion of In adatoms from the substrate can be safely neglected because the InP NW stem is around 1.2 μm long. According to Figure 2, the length growth rate is higher (around 28 nm/min) for the shortest growth time of 30 min, and decreases to nearly 15 nm/min for 60 min and 90 min. The direct impingement is constant throughout the InSb growth, hence a faster elongation at the beginning of growth can be associated with a larger diffusion flux of In adatoms probably from both InP and InSb sidewalls. The latter is characterized by an effective diffusion length *λ_In_*. When the length of InSb segment becomes greater than *λ_In_*, the contribution from sidewall diffusion decreases [24]. The diffusivity of In adatoms on the InSb sidewalls is expected to be limited by a significant radial growth. Therefore, after NF length reaches the diffusion length *λ_In_*, the axial growth rate becomes proportional to the direct flux of In atoms impinging the catalyst and the upper part of the NF and hence independent of the growth time. As for the pitch dependence of the NF length, Figure 2 shows that shorter NFs (square symbols) are obtained for 500 nm pitch, at all growth times. Lower axial growth rates for smaller pitches should be due to shadowing or competition between the neighboring NFs for the material flux. For larger pitches (a ≥ 700 nm) the NF lengths are almost independent of the pitch, indicating no competition above this threshold.

Time evolution of the length of InSb NFs can be quantified using the following model. For short length $L<\lambda_{In}$, In atoms are collected from the entire length of the NF, while for $L>\lambda_{In}$ the In sidewall diffusion is effective only in the upper part of the NF of length $\lambda_{In}$, yielding

(1) $$\frac{dL}{dt}=AL+B,\;L\leq \lambda_{In},\;\frac{dL}{dt}=C\lambda_{In}+B,\;L>\lambda_{In}.$$

Here, A and C stand for the efficiencies of In adatom collection from the NW sidewalls, which are different in different stages of growth according to Ref. [36], while B describes the direct impingement of In atoms onto the catalyst surface. The parameter *A* increases with the pitch due to the shadowing effect on the NW/NF sidewalls. Solutions are given by:

(2) $$L=\frac{B}{A}\left(e^{At}-1\right),\;L\leq \lambda_{In};\;L=\lambda_{In}+\left(C\lambda_{In}+B\right)\left(t-t_{0}\right),\;L>\lambda_{In},$$

where $t_{0}$ is the moment in time at which the NF length reaches $\lambda_{In}$. According to Equation (2), the NF length increases first exponentially and then linearly with time. The line in Figure 2 shows a good fit for the length of InSb NFs obtained from Equation (2) with $\lambda_{In}=$ 724 nm at a= 500 nm. This fit is obtained with A= 0.158 min^−1^, B= 1 nm/min, and C= 0.013 min^−1^. The fit obtained using this simple model reproduces the non-linear evolution of the NF length with time. A more detailed analysis requires further investigation and will be presented elsewhere.

The VS radial growth rate of the InSb NFs, which controls the NF width and thickness, is 10 times lower than the VLS axial growth rate. According to Figure 3a,b, the VS growth rate of thickness and width is more sublinear for the smallest pitch of 500 nm, in which case the thickness is almost constant after 60 min. For the largest pitch of 1500 nm, both thickness and width increase almost linearly with time.

To understand these trends for the NF thickness and width, we propose a model which takes into account the direct and re-emitted fluxes of Sb, and the shadowing effect [28]. The re-emitted flux originates from scattering of Sb atoms from the mask surface and NF/NW sidewalls, as in Ref. [37] for As. We assume that (i) a certain amount of Sb atoms will contribute to the width growth at any time from the direct flux; (ii) the re-emitted Sb flux can be almost fully shadowed; (iii) the re-emitted flux is not directional; and (iv) the shadowing effect increases for smaller pitches.

The model geometry is shown in Figure 3c. We restrict the study to a simplified 2D geometry, where the width W corresponds to the most extended part of the NF. If the substrate were rotated during growth of NFs, the width $W_{0}$ and thickness T would be related simply as $W_{0}=\left(2/\sqrt{3}\right)T$ from regular hexahedral geometry. Without substrate rotation, the width becomes greater,

(3) $$W=\frac{2}{\sqrt{3}}T+\Delta W$$

where ΔW is the additional width of a NF due to the direct Sb flux impinging only two of the six side facets (see Figure 3c). As generally recognized in the growth modeling of free-standing III–V NWs and NFs [18,24,25,26,27,28,29,30,32,33,35,36,37,38,39], group III atoms may diffuse along and around the NW, while group V species are not diffusive due to their high volatility. In our case, the diffusion length of In adatoms along the sidewalls is more than 700 nm as discussed above. Therefore, diffusive In atoms can reach the back side of the NF by sidewall diffusion across the corners, while Sb atoms can impinge onto the back side only from re-emitted flux. Volume diffusion of In should not be effective at a low growth temperature of 365 °C [26,28]. According to Equation (3), the growth rate $dW/dt=\left(2/\sqrt{3}\right)dT/dt$ is due to re-emitted Sb flux, while dΔW/dt originates from the direct Sb flux in the absence of substrate rotation and leads to asymmetric geometry of the NF. We can thus write

(4) $$\frac{dT}{dt}=v_{r}$$

where $v_{r}$ is the re-emitted flux of Sb atoms. According to Equation (4), the NF thickness increases only due to re-emitted flux, which is not directional but rather originates from Sb vapor surrounding the NFs.

Without this re-emitted flux of Sb atoms in the absence of substrate rotation, the NFs would grow only in the direction opposing the Sb flux and the thickness *T* would stay constant. Using Equation (4) in Equation (3) along with ΔW/dt=v, with v as the direct flux of Sb atoms, we obtain

(5) $$\frac{dW}{dt}=\frac{2}{\sqrt{3}}v_{r}+v$$

The re-emitted flux of Sb *v_r_* is shadowed by the neighboring NF/NW structures. The shadowing effect must increase for smaller pitches a and longer growth times t [38,39]. In the first approximation, the re-emitted flux can be expressed as

(6) $$v_{r}=\varepsilon v\left(1-\frac{kt}{a}\right),\frac{kt}{a}<1,\;\mathrm{or}\;0,\;\frac{kt}{a}\geq 1$$

where ε gives the ratio of re-emitted over direct flux in the absence of shadowing and k is a constant. The re-emitted flux becomes fully shadowed after a certain time corresponding to saturation of the NF thickness. Using Equation (6) in Equations (4) and (5) and integrating, we get

(7) $$T=T_{0}+\varepsilon vt-\frac{k\varepsilon v}{2a}t^{2},\;t<\frac{a}{k};\;T=T_{0}+\frac{\varepsilon va}{2k}=T_{*},\;t\geq \frac{a}{k},\;W=\frac{2}{\sqrt{3}}T+vt,t<\frac{a}{k};W=\frac{2}{\sqrt{3}}T_{*}+\frac{va}{k}+v\left(t-\frac{a}{k}\right),t\geq \frac{a}{k}.$$

Lines in Figure 3a,b show the fits for the largest and smallest pitches, a= 1500 nm and 500 nm, obtained from Equation (7) at $D_{0}=W_{0}=$ 38 nm, v= 2.4 nm/min according to the data, ε= 0.625, and k=4 nm/min. With these fitting parameters, we are able to reproduce the main trends such as saturation of the NF thickness for the smallest pitch and gradual increase in the NF width regardless of the pitch. The fits are not perfect and cannot be better due to the scattered data and simplifications of the model. However, we can expect that the aspect ratio W/T will increase for longer growth times and that the enlargement of the NF thickness can be decoupled from the increase in their width as soon as the re-emitted flux is completely suppressed, yielding a more asymmetric geometry of InSb NFs.

Summarizing the modeling results, the elongated quasi-2D shape of InSb NFs originates from the directional Sb flux in the absence of substrate rotation, where the NFs preferentially grow in the direction opposing the flux. Additional flux of Sb atoms re-emitted from the masked substrate surface and from the NF sidewalls leads, however, to the enlargement of the NF thickness. Under a given set of growth conditions, the NF thickness should saturate at a certain time, which is shorter for a smaller pitch or higher surface density of InP NW stems.

## 4. Conclusions

In conclusion, quasi-2D InSb NFs were grown by CBE in regular arrays using InP NW templates synthesized on patterned SiO_2_/InP(111)B substrates. Asymmetric geometry of the NFs was achieved by stopping the substrate rotation and properly aligning the sample with the impingent beam. A simplified 2D growth model was presented which allowed for semi-quantitative description of the NF morphology as a function of the growth time and pitch. Although the real hexagonal shape of the NFs was approximated to a simpler rectangular one, we were able to correctly reproduce the main trends of the morphological evolution of the array of NFs in terms of their length, width and thickness versus growth time and pitch. The pitch-dependent shadowing effect for the re-emitted Sb flux was identified as the key process influencing the morphological evolution, in particular the width-over-thickness ratio. According to the model, the NF thickness should saturate after a time corresponding to the full shadowing of Sb flux re-emitted from the masked substrate surface, while the NF width continues to grow in the direction opposing the Sb flux in the absence of substrate rotation. Therefore, larger width-to-thickness ratios are expected to be realized for longer growth times and smaller pitches. Overall, these findings can be used to tune the InSb NF morphology and can probably be extended to other material systems. A better understanding of the growth mechanisms of the NFs can be obtained by performing real-time studies such as in situ observations with a transmission electron microscope. We plan to perform more experiments and modeling to access the NF morphology for longer growth times and develop a more detailed understanding of the entire growth process in the real 3D geometry, particularly the regimes corresponding to the highest aspect ratios of the NFs.

## Data Availability

Data is contained within the article or Supplementary Material.

## Supplementary Materials

The following supporting information can be downloaded at: https://www.mdpi.com/article/10.3390/nano12071090/s1. Figure S1. Low-magnification SEM image of InSb NFs.
