# Supplementary material for: Understanding the Morphological Evolution of InSb Nanoflags Synthesized in Regular Arrays by Chemical Beam Epitaxy
Source: Nanomaterials (Basel). 2022 Mar 26;12(7):1090. doi: 10.3390/nano12071090 (PMC9000652; doi:10.3390/nano12071090)
Supplement: Supplementary file 1 [file nanomaterials-12-01090-s001.zip › nanomaterials-1611324-supplementary.pdf]

nanomaterials

Supplementary Materials

Understanding the Morphological Evolution of InSb Nanoflags Synthesized in Regular Arrays by Chemical Beam Epitaxy

Isha Verma [1], Valentina Zannier [1,*], Vladimir G. Dubrovskii [2], Fabio Beltram [1] and Lucia Sorba [1]

[1] NEST, Scuola Normale Superiore and Nanoscienze-CNR, Piazza San Silvestro 12, I-56127 Pisa, Italy; isha.verma@sns.it (I.V.); fabio.beltram@sns.it (F.B.); lucia.sorba@nano.cnr.it (L.S.)
[2] Faculty of Physics, St. Petersburg State University, Universitetskaya Emb. 13B, 199034 St. Petersburg, Russia; dubrovskii@mail.ioffe.ru
* Correspondence: valentina.zannier@nano.cnr.it

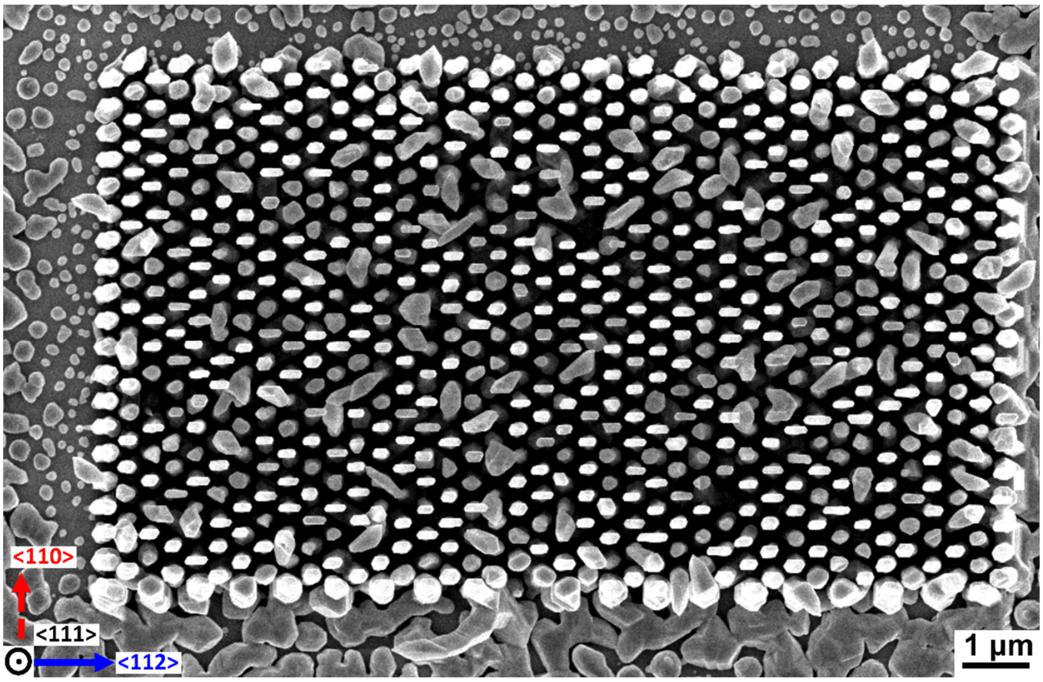

Figure S1. Low magnification top-view SEM image of InP-InSb heterostructure NFs grown for 60 min in the pattern having a pitch a of 700 nm. The yield of straight InSb NFs is ~ 87%.
